# COVID-19 pandemic and distinct patterns of psychotic outbreaks

**DOI:** 10.47626/2237-6089-2020-0188

**Published:** 2021-12-10

**Authors:** Carlos Gustavo Costardi, Daniel A. Cavalcante, Marcos Antônio Macêdo, Raphael de O Cerqueira, Maria Carolina Rios, Cristiano Noto, Ary Gadelha

**Affiliations:** 1 Laboratório Interdisciplinar de Neurociências Clínicas Escola Paulista de Medicina Universidade Federal de São Paulo São Paulo SP Brazil Laboratório Interdisciplinar de Neurociências Clínicas (LiNC), Escola Paulista de Medicina – Universidade Federal de São Paulo (EPM-UNIFESP), São Paulo, SP, Brazil.; 2 Grupo de Atenção às Psicoses Iniciais Departamento de Psiquiatria EPM-UNIFESP São Paulo SP Brazil Grupo de Atenção às Psicoses Iniciais (GAPi), Departamento de Psiquiatria, EPM-UNIFESP, São Paulo, SP, Brazil.; 3 Programa de Esquizofrenia Departamento de Psiquiatria EPM-UNIFESP São Paulo SP Brazil Programa de Esquizofrenia (PROESQ), Departamento de Psiquiatria, EPM-UNIFESP, São Paulo, SP, Brazil.; 4 Centro de Pesquisa e Inovação em Prevenção de Transtornos Mentais e Uso de Álcool e Outras Drogas EPM-UNIFESP São Paulo SP Brazil Centro de Pesquisa e Inovação em Prevenção de Transtornos Mentais e Uso de Álcool e Outras Drogas (CEPIPREV), EPM-UNIFESP, São Paulo, SP, Brazil.

There is concern about the impact of the coronavirus disease 2019 (COVID-19) pandemic on mental health and, more specifically, on the incidence of psychotic disorders. However, limited evidence is available to examine predictions and to plan preventive measures.^1^

We reviewed the clinical data of patients diagnosed with a psychotic disorder and presenting a chief complaint related to the COVID-19 pandemic. All patients were evaluated at the psychiatric emergency unit of Universidade Federal de São Paulo (UNIFESP), between March and August 2020, in São Paulo, Brazil. Confidentiality of all subjects was preserved, and the study was approved by the research ethics committee of the UNIFESP (CAAE 33124620.0.0000.5505). Sociodemographic characteristics, clinical features, diagnostic hypotheses, and complementary information are presented in Table 1.

**Table 1:** Sociodemographic and clinical characteristics

	Case A	Case B	Case C	Case D	Case E	Case F
Sociodemographic characteristics						
Age	26	45	48	11	65	34
Gender	Female	Female	Male	Female	Female	Female
Marital status	Single	Unavailable	Single	Single	Married	Single
Education	Higher	Primary	Higher	Primary	Unavailable	Unavailable
Psychopathology						
Delusions	Yes	Yes	Yes	Yes	Yes	Yes
Hallucinations	No	No	Yes	No	No	No
Agitated or disorganized behavior	Yes	No	Yes	Yes	No	Yes
Clinical history related to COVID-19 pandemic	Patient said she could cure COVID-19 and mentioned that the virus was the apocalypse	Patient believed that her neighbors had passed information about her COVID-19 infection to the government	Patient presented fixed idea of being infected with the virus, evolving with episodes of agitation	Excessive fear of COVID-19, asking for silence at home, so that neighbors could not hear her family	Patient said that everyone would die from COVID-19 and reported that the coronavirus came out of her urine	Patient repeatedly said the coronavirus would catch her, presenting disorganized behavior
Clinical features						
History of psychotic symptoms	No	No	No	No	Yes (1 previous episode)	Yes (1 previous episode)
History of psychiatric treatment	Yes	No	No	No	Yes	Yes
History of substance use	Alcohol	No	Alcohol	No	No	No
Family history of mental disorders	Yes	Yes	No	No	Yes	Yes
Previous COVID-19 infection	No	Yes	No	No	No	No
Diagnostic hypothesis*	Bipolar I disorder	Brief psychotic disorder	Brief psychotic disorder	Brief psychotic disorder	Brief psychotic disorder	Schizophrenia
Suicidal ideation or behavior	No	No	Yes	No	No	No
Need for psychiatry hospitalization	Yes	No	Yes	No	No	Yes
	**Case G**	**Case H**	**Case I**	**Case J**	**Case K**	**Case L**
Sociodemographic characteristics						
Age	21	58	57	45	26	43
Gender	Male	Male	Female	Female	Female	Male
Marital status	Single	Single	Married	Single	Single	Married
Education	Secondary	Higher	Unavailable	Higher	Secondary	Primary
Psychopathology						
Delusions	Yes	Yes	Yes	Yes	Yes	Yes
Hallucinations	Yes	No	Yes	No	Yes	Yes
Agitated or disorganized behavior	Yes	Yes	Yes	Yes	Yes	Yes
Clinical history related to COVID-19 pandemic	Patient said he had influenced God and Lucifer to start the COVID-19 pandemic	Patient presented delusions about the end of the world by the COVID-19 pandemic	Patient felt guilty for creating the COVID-19 pandemic	Patient claimed to have the cure for coronavirus, and that she could end the COVID-19 pandemic	Patient stated that the coronavirus meant the end of the world and that God was returning	Patient said he was sent by God to help in the pandemic and that he had the gift of healing
Clinical features						
History of psychotic symptoms	Yes (multiple episodes)	Yes (multiple episodes)	Yes (multiple episodes)	Yes (multiple episodes)	Yes (multiple episodes)	Yes (multiple episodes)
History of psychiatric treatment	Yes	Yes	Yes	Yes	Yes	Yes
History of substance use	Cannabis/Cocaine	Unavailable	No	No	No	No
Family history of mental disorders	No	Unavailable	Yes	No	No	Yes
Previous COVID-19 infection	No	No	No	No	No	No
Diagnostic hypothesis*	Schizophrenia	Bipolar I disorder	Schizophrenia	Bipolar I disorder	Schizophrenia	Bipolar I disorder
Suicidal ideation or behavior	No	No	Yes	No	No	No
Need for psychiatry hospitalization	Yes	Yes	Yes	Yes	Yes	Yes

COVID-19 = coronavirus disease 2019.

* According to the Diagnostic and Statistical Manual of Mental Disorders, 5th edition (DSM-5).^2^

Of 12 individuals, 4 (33.3%) presented new-onset psychosis, while 8 had a previous history of psychotic symptoms. Among new-onset patients, the diagnosis of brief psychotic disorder, according to the Diagnostic and Statistical Manual of Mental Disorders, 5th edition (DSM-5),^2^ was the most prevalent one (75%). Eight patients (66.6%) were hospitalized due to severe psychomotor agitation and/or suicidal ideation. Seven of them received a diagnosis of schizophrenia or bipolar I disorder and one received a diagnosis of brief psychotic disorder. All cases presented delusions or hallucinations with content related to the pandemic.

Psychotic symptoms can be understood as a cognitive scheme developed to explain an aberrant salience experience,^3^ and they commonly incorporate external (recent or stressful) events,^4^ such as the COVID-19 pandemic. Psychosis may emerge via two non-competing pathways in the context of the pandemic. First, there is the psychological distress leading to brief psychotic reactions or anticipating disease onset/relapse in those more genetically vulnerable. Second, psychosis may be a result of the direct effect of the viral infection on the brain, including post-viral presentations and treatment-related complications such as steroid-induced psychosis.^5^

Only one individual had a history of COVID-19 infection, but there were no signs of clinical impairment related to viral infection or complications. Three out of four patients with new-onset psychosis did not present prodromal features of psychotic disorders, i.e., they presented delusion but neither gross disorganization nor negative symptoms. Also, these patients had an atypical age of onset for first-episode psychosis,^6^ and presented lower psychiatric risks or need for hospitalization, as opposed to patients with a previous history of psychotic symptoms. Such features suggest a more benign evolution and demand closer follow-up to define the need to maintain antipsychotic treatment and titration, avoiding unnecessary harm due to treatment.^7^

In conclusion, we observed two patterns of psychotic disorders related to the psychological distress caused by the COVID-19 pandemic: 1) brief, apparently mild new-onset cases; and 2) relapse of previously diagnosed patients. The reduced number of cases directly associated with viral infection can be explained by the study design, which included patients who directly sought psychiatric care. Moreover, it can suggest that psychological distress represents a higher burden to psychotic outcomes amid the COVID-19 pandemic, but this needs to be addressed in larger, representative samples. Further epidemiological studies are needed to assess a possible increased risk for psychotic disorders related to the pandemic, and follow-up studies can help to better understand the evolution and clinical outcome of the related disorder.
